# Using Out-of-Hospital Cardiac Arrest (OHCA) and Cardiac Arrest Hospital Prognosis (CAHP) Scores with Modified Objective Data to Improve Neurological Prognostic Performance for Out-of-Hospital Cardiac Arrest Survivors

**DOI:** 10.3390/jcm10091825

**Published:** 2021-04-22

**Authors:** Hogul Song, Jungsoo Park, Yeonho You, Hongjoon Ahn, Insool Yoo, Seungwhan Kim, Jinwoong Lee, Seung Ryu, Wonjoon Jeong, Yongchul Cho, Changshin Kang

**Affiliations:** 1 Department of Emergency Medicine, Chungnam National University Hospital, 282, Munhwa-ro, Jung-gu, Daejeon 35015, Korea; songhg@cnuh.co.kr (H.S.); yyh1003@hanmail.net (Y.Y.); jooniahn@hanmail.net (H.A.); mdinsool@cnuh.co.kr (I.Y.); emfire@cnuh.co.kr (S.K.); emd93@hanmail.net (J.L.); rs0505@cnuh.co.kr (S.R.); gardenjun@hanmail.net (W.J.); boxter73@naver.com (Y.C.); changsiny@naver.com (C.K.); 2 Department of Emergency Medicine, College of medicine, Chungnam National University, Daejeon 35015, Korea

**Keywords:** out-of-hospital cardiac arrest, prognosis, outcome, prediction score

## Abstract

This study aimed to determine whether accuracy and sensitivity concerning neurological prognostic performance increased for survivors of out-of-hospital cardiac arrest (OHCA) treated with targeted temperature management (TTM), using OHCA and cardiac arrest hospital prognosis (CAHP) scores and modified objective variables. We retrospectively analyzed non-traumatic OHCA survivors treated with TTM. The primary outcome was poor neurological outcome at 3 months after return of spontaneous circulation (cerebral performance category, 3–5). We compared neurological prognostic performance using existing models after adding objective data obtained before TTM from computed tomography (CT), magnetic resonance imaging (MRI), and biomarkers to replace the no-flow time component of the OHCA and CAHP models. Among 106 patients, 61 (57.5%) had poor neurologic outcomes. The area under the receiver operating characteristic (AUROC) curve for the OHCA and CAHP models was 0.89 (95% confidence interval (CI) 0.81–0.94) and 0.90 (95% CI 0.82–0.95), respectively. The prediction of poor neurological outcome improved after replacing no-flow time with a grey/white matter ratio measured using CT, high-signal intensity (HSI) on diffusion-weighted MRI (DWI), percentage of voxel using apparent diffusion coefficient value, and serum neuron-specific enolase levels. When replaced with HSI on DWI, the AUROC and sensitivity of the OHCA and CAHP models were 0.96 and 74.5% and 0.97 and 83.8%, respectively (100% specificity). Prognoses concerning neurologic outcomes improved compared with existing OHCA and CAHP models by adding new objective variables to replace no-flow time. External validation is required to generalize these results in various contexts.

## 1. Introduction

Despite advances in cardiac arrest (CA) resuscitation, the survival rate remains low (out-of-hospital cardiac arrest (OHCA), 12%; in-hospital cardiac arrest, 25%) [1]. Many of these deaths result from the withdrawal of life-sustaining treatment (WLST) due to predicted poor neurological outcomes [2]. Current guidelines recommend neurologic prognosis 72 h post-CA; however, WLST earlier than 72 h has been noted and shown to increase the mortality rate among patients who had survived after CA [3].

Therefore, in the initial stages before initiating TTM, it is important to provide a patient’s family and the treating physician with an accurate neurologic prognosis to prevent inappropriate WLST or to avoid prolonged treatment for patients with no chance of neurologically meaningful survival. Moreover, during a pandemic such as the current coronavirus disease pandemic, when abundant critical care resources are needed, early and accurate prognosis is essential to ensure the appropriate distribution of limited medical resources.

Several CA-specific risk scores are used to predict neurologic outcomes for post-CA survivors before TTM, such as out-of-hospital cardiac arrest (OHCA), cardiac arrest hospital prognosis (CAHP), and C-GRApH score [4,5,6]; however, these CA-specific risk scores have several limitations. First, in patients with unwitnessed collapse, no-flow time (time from collapse to cardiopulmonary resuscitation (CPR)) with an unknown exact value is included in the equation. Second, the OHCA and CAHP scores do not provide a definitive indication as to whether TTM had a significant effect on patient prognosis. Third, these prediction tools predict a poor neurological outcome; therefore, when the false-positive rate is 0%, it is a good prediction method to show high sensitivity, but these scores still have low sensitivity. Recently, various studies have focused on improving these CA-specific risk scores by adding new factors or by comparing each prediction model with other models [7,8,9,10]. However, more accurate prediction models are needed to minimize the risk of erroneous prognostication concerning poor neurological outcomes.

This study aimed to determine whether neurological prognostic accuracy and sensitivity increased through replacing no-flow time information with data obtained from brain magnetic resonance imaging (MRI), computed tomography (CT), or biochemical indicators (neuron-specific enolase (NSE)), to more objective representing hypoxic ischemic encephalopathy occurring before TTM as a variable of the CA-specific risk score.

## 2. Patients and Methods

### 2.1. Study Design and Patients

In this retrospective single-center observation cohort study, we analyzed prospectively collected data from non-traumatic comatose OHCA adult survivors treated with TTM at Chungnam National University Hospital (CNUH), Daejeon, Korea, from May 2018 to October 2020. This study was approved by the CNUH Institutional Review Board. CNUH is a tertiary teaching hospital in Korea that provides medical services to >50,000 emergency department patients annually.

The inclusion criteria were patients (age, >18 years) with non-traumatic CA, who had been resuscitated and treated with TTM. The exclusion criteria were patients (i) who were ineligible for TTM (i.e., those with brain hemorrhage, active bleeding, refused further treatment with a do not resuscitate order, known terminal illness, poor pre-arrest neurological status, and those who had undergone extracorporeal membrane oxygenation (ECMO)); (ii) who failed to maintain a temperature of 33 °C during TTM due to unstable hemodynamics; (iii) whose TTM initiation was performed 6 h after return of spontaneous circulation (ROSC); (iv) who had insufficient laboratory data obtained <6 h after ROSC; or (v) who had missing information at the time of CA or CPR.

### 2.2. Target Temperature Management Protocol

All patients who were comatose due to non-traumatic OHCA and eligible for TTM received treatment according to recent resuscitation guidelines [11]. TTM was started within 6 h after ROSC using a feedback-controlled surface cooling device (Arctic Sun^®^; Medivance Corp., Louisville, Co., KY, USA). A target temperature of 33 °C was maintained for 24 h and monitored with a bladder prove. After completing a 24 h duration TTM maintenance phase, each patient was carefully rewarmed to 37 °C at a rate of 0.25 °C per hour. During TTM, all patients received sedatives and a neuromuscular blocking agent.

### 2.3. Brain Imaging and Biochemical Indicators

CT scans were used to measure the grey/white matter ratio (GWR). High-signal intensity (HSI) on diffusion-weighted imaging (DWI) and voxel-based apparent diffusion coefficient (ADC), measured using MRI, were used for brain imaging. Brain CT scans had been taken using a 64-channel system (SOMATOM Sensation 64, Siemens Healthineers, Munich, Germany). Hounsfield units were recorded at the caudate nucleus (CN), posterior limb of the internal capsule (PIC), corpus callosum (CC), putamen (P), and thalamus (T). GWR was defined as the mean value of 6 ratios: CN/CC, P/CC, T/CC, CN/PIC, P/PIC, and T/PIC [12,13]. Brain DWI was performed using a 3T scanner (Achieva, Philips Healthcare, Amsterdam, The Netherlands) before TTM. The imaging protocol and analysis used were the same as those published previously [14,15,16]. Previous studies used the percentage of voxels (PV) below different ADC thresholds and reported that PV 400 (percentage of voxels <400 × 10^−6^ mm^2^/s) showed the highest odds ratio value when predicting the poor prognosis of a patient [14]; therefore, we also used PV 400.

NSE levels were measured in serum samples obtained by venepuncture between brain imaging and TTM initiation. An electrochemiluminescence immunoassay kit (COBAS^®^ e801, Roche Diagnostics, Basel, Switzerland) was used to measure the NSE level (NSE measurement range, 0.1–300 ng/mL (normal value, <16.3 ng/mL)) [17].

### 2.4. Outcomes and Data Collection

The primary outcome in this study was poor neurological outcome 3 months after ROSC. The neurological outcome was measured using the Glasgow–Pittsburgh cerebral performance category (CPC) scale, which was determined through face-to-face interviews or structured telephone interviews. The CPC level was categorized into 5 levels, with CPC levels 1 (good performance) and 2 (moderate disability) classified as good neurological status. CPC levels 3 (severe disability), 4 (vegetative state), and 5 (death, brain death) were classified as poor neurological status.

The following data were obtained from electronic medical records by an investigator who was blinded to the study objective: age, sex, comorbidities, witnessed status, location of primary CPR, bystander CPR, initial rhythm (shockable vs. non-shockable), CA etiology, time from collapse to first CPR (no-flow time), time from CPR to ROSC (low-flow time), injected adrenaline (epinephrine) dosage until ROSC, and any received intervention prior to TTM. The first available laboratory data after ROSC (levels concerning arterial pH, lactate, albumin, neutrophil gelatinase-associated lipocalin (NGAL), NSE, creatinine kinase myocardial band (CK-MB), troponin I, white blood cells, C-reactive protein, procalcitonin, and interleukin-6) were collected. In addition, PaCO_2_ levels in the first 6 h after ROSC were analyzed with a time-weighted average (TWA). The TWA was analyzed in a manner consistent with previously published methods of calculating exposure over time [18]. NSE level data were collected on days 0, 1, 2, and 3 after ROSC.

We calculated three CA-specific risk scores (OHCA, CAHP, and C-GRApH) following standard practice in relation to these scores. The OHCA score includes initial rhythm, no-flow and low-flow times, and blood lactate and creatinine levels [4]. The CAHP score model includes age, location of arrest, initial rhythm, no-flow and low-flow times, pH level, and adrenaline dose [5]. The C-GRApH score model includes known coronary artery disease pre-OHCA, glucose level, initial rhythm, age, and arterial pH level [6]. We defined modified OHCA (M-OHCA) and CAHP (M-CAHP) as the prediction models estimated from probability value of pre-existing variables without considering standard logistic regression b-coefficients for scoring. To suppress a bias from inaccurate no-flow time, the no-flow time was substituted into an objective value obtained from imaging study or serum biomarker. Based on the substituted objective variables (e.g., GWR of CT, HSI on DWI, PV 400 of ADC, and the serum NSE level) were defined as follows: M-OHCA_GWR_, M-OHCA_HSI_, M-OHCA_PV400_, M-OHCA_NSE_, M-CAHP_GWR_, M-CAHP_HSI_, M-CAHP_PV400_, and M-CAHP_NSE_.

### 2.5. Statistical Analysis

Continuous data are presented as mean ± standard deviation or median with interquartile range (IQR) values using a normality test, and categorical variables were presented as frequencies and percentages. Mann–Whitney U or independent t-test was used to determine differences between two groups of continuous or ordinal variables, respectively. Fisher’s exact or chi-square test was used to compare categorical data.

We used the receiver operating characteristic (ROC) curve to determine the predicted performance, and the Delong test was used to compare the area under the ROCs (AUC). The sensitivity to predict poor neurological outcome at 3 months after ROSC was calculated when 100% specificity was maintained. AUC values for M-OHCA and M-CAHP models were estimated in two steps. A probability value of the modified model components was first obtained through binary logistic regression analysis. We then conducted ROC analysis using this probability as a test variable. To test the alternative superiority of brain imaging and biomarkers compared to the OHCA and CAHP original models, the no-flow time was replaced by GWR of CT, HSI of DWI, PV 400 and NSE of ADC, and then compared the predictive performance. Statistical analyses were performed using SPSS, version 21.0 (Chicago, IL, USA) and MedCalc 15.2.2 (MedCalc software, Mariakerke, Belgium) software. *p* values ≤ 0.05 were considered statistically significant.

## 3. Results

### 3.1. Patient Characteristics

**Figure 1:**
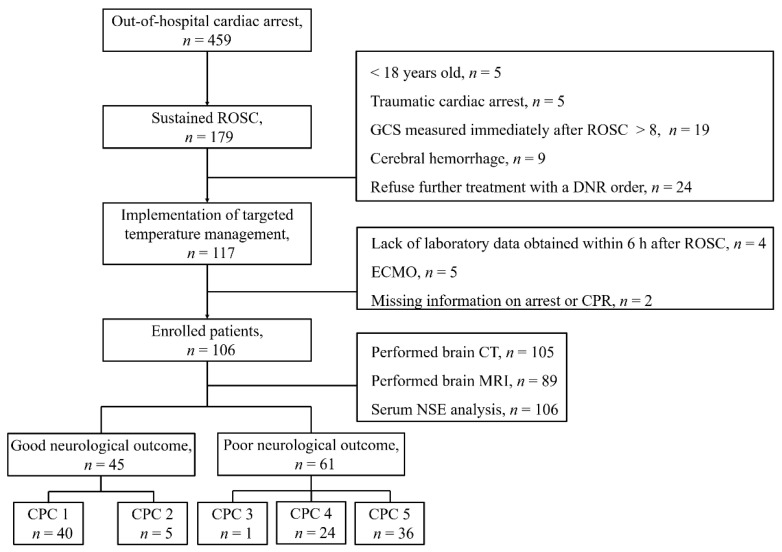
Flow diagram showing the selection of study patients.

In total, 117 adult patients with non-traumatic OHCA were treated with TTM during the study period. Of them, 11 were excluded for the following reasons: insufficient laboratory data obtained within 6 h after ROSC (*n* = 4), having received ECMO (*n* = 5), and missing information concerning the etiology of CA or CPR (*n* = 2, Figure 1). At 3 months after ROSC, 45 (42.5%) patients were found to be in the good neurological outcome group, whereas 61 (57.5%) were in the poor neurological outcome group. Data of patient demographics, OHCA characteristics, laboratory data, brain imaging results, and CA-specific risk scores, stratified according to the neurological outcome at 3 months, are shown in Table 1.

### 3.2. Prognostic Performance of Each Method

Table 2 shows the prognostic performance of each method 3 months after ROSC. AUC values concerning the OHCA, CAHP, and C-GRApH scores were found to be 0.86 (95% confidence interval (CI) 0.78–0.92), 0.80 (95% CI 0.71–0.87), and 0.70 (95% CI 0.60–0.78), respectively, in our cohort. Of these, the OHCA score showed a significantly higher predictive performance than the C-GRApH score (P = 0.001). Among the biomarker and brain images, HSI on DWI showed the strongest prognostic performance, followed by NSE, PV 400 of ADC, and GWR of CT. The sensitivity of each method for predicting poor neurological outcome with 100% specificity was 69.2%, 47.5%, 40.4%, and 13.3%, respectively.

### 3.3. Prognostic Performance Comparison Using Modified OHCA and CAHP Models

**Figure 2:**
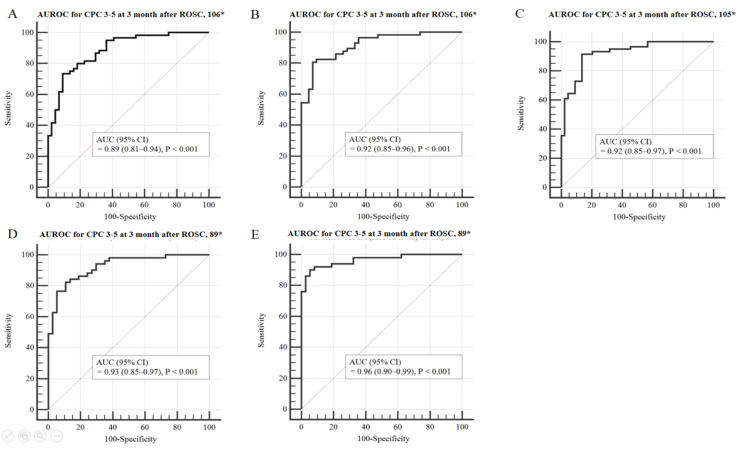
Area under the curve (AUC) to predict poor neurological outcome 3 months post-OHCA in the modified OHCA model. The ROC curve for the M-OHCA model is shown in panel (**A**); the ROC curve for the M-OHCA_NSE_ model is shown in panel (**B**); the ROC curve for the M-OHCA_GWR_ model is shown in panel (**C**); the ROC curve for the M-OHCA_PV400_ model is shown in panel (**D**); the ROC curve for the M-OHCA_HSI_ model is shown in panel (**E**). Abbreviations: OHCA, out-of-hospital cardiac arrest; ROC, receiver operating characteristics; NSE, neuron-specific enolase; GWR, grey/white matter ratio; PV, percentage of voxel; HSI, high signal intensity; CI, confidence interval. * Number of patients included in the analysis.

**Figure 3:**
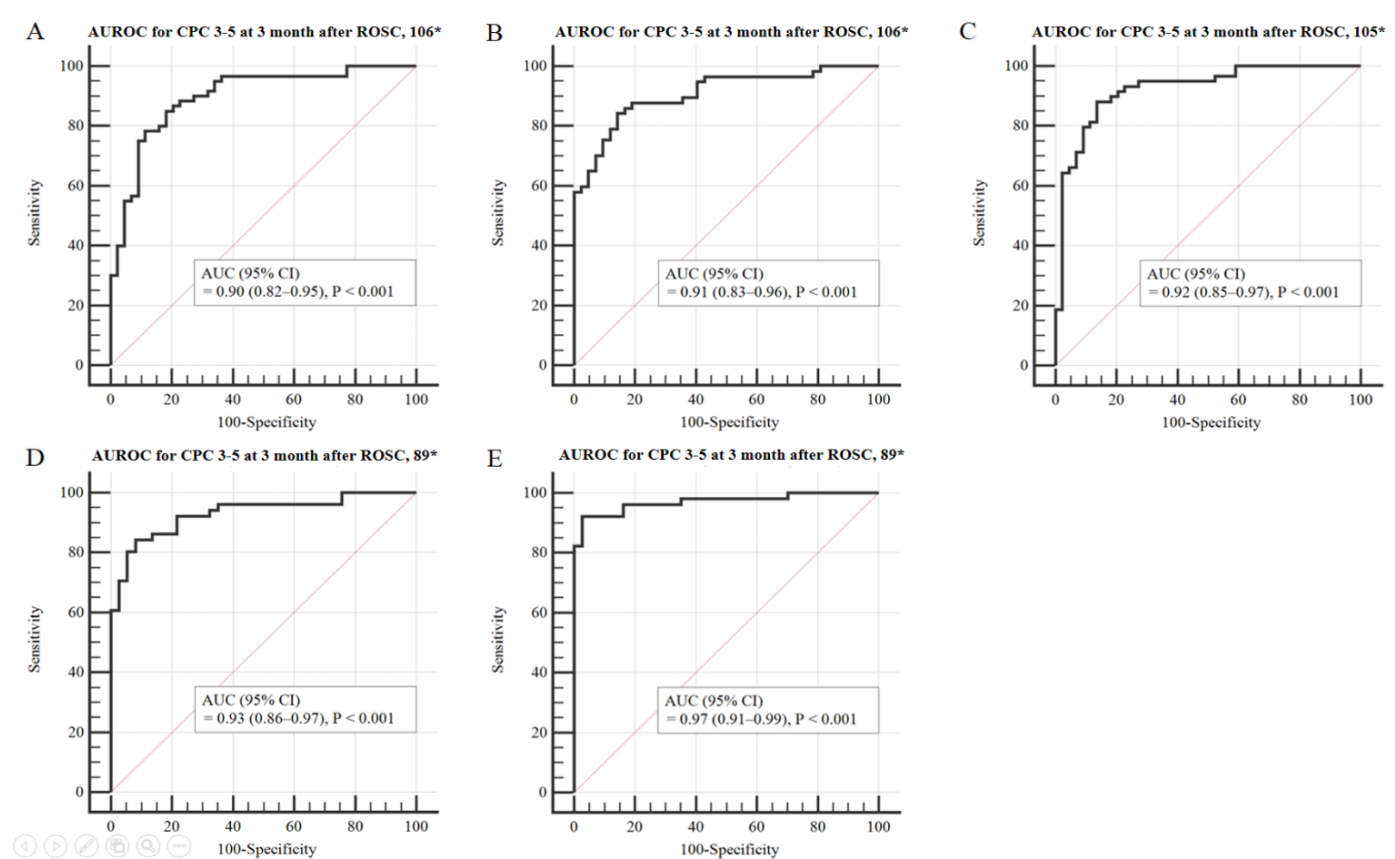
Area under the curve (AUC) to predict poor neurological outcome 3 months post-OHCA in the modified CAHP model. The ROC curve for the M-CAHP model is shown in panel (**A**); the ROC curve for the M-CAHP_NSE_ model is shown in panel (**B**); the ROC curve for the M-CAHP_GWR_ model is shown in panel (**C**); the ROC curve for the M-CAHP_PV400_ model is shown in panel (**D**); the ROC curve for the M-CAHP_HSI_ model is shown in panel (**E**). Abbreviations: CAHP, cardiac arrest hospital prognosis; ROC, receiver operating characteristics; NSE, neuron-specific enolase; GWR, grey/white matter ratio; PV, percentage of voxel; HSI, high signal intensity; CI, confidence interval. * Number of patients included in the analysis.

Table 3 shows a comparison of prognostic performance using the modified OHCA and CAHP models. Prognosis prediction using the probability value from either the OHCA or CAHP model was higher than that of the existing score model (0.86 vs. 0.89, 0.80 vs. 0.90, respectively). However, at 100% specificity, the sensitivity remained low at 33.3% and 30.0%, respectively. Compared with using the M-OHCA model, the M-OHCA_NSE_, M-OHCA_GWR_, M-OHCA_PV400_, and M-OHCA_HSI_ models showed gradual improvements in terms of prognostic performance (Figure 2). Furthermore, compared with using the M-CAHP model, the M-CAHP_NSE_, M-CAHP_GWR_, M-CAHP_PV400_, and M-CAHP_HSI_ models showed gradual improvements in terms of prognostic performance (Figure 3). The M-OHCA_HSI_ and M-CAHP_HSI_ models showed significant improvement in terms of prognostic performance compared to the conventional modified OHCA and CAHP models, respectively (all *p* = 0.01). Moreover, both models showed 74.5% and 82.4% at the highest sensitivity, respectively, when the specificity was 100%.

## 4. Discussion

In this retrospective single-center observation cohort study of a South Korean population, in the OHCA and CAHP models, when objective no-flow time data were replaced with data concerning HSI on DWI, PV 400 of ADC, GWR of CT, and NSE levels, both prediction and sensitivity were further improved. Of these that were modified, the M-OHCA_HSI_ and M-CAHP_HSI_ models showed significantly higher AUC values and sensitivity than the conventional model and other combination models.

The results of this study are not intended to be used to influence premature WLST decision-making. Rather, we anticipate that the results of our study could help the treating physicians and guardians of patients with a poor prognosis in making more informed decisions concerning critical interventions such as continuous renal replacement therapy or extracorporeal membrane oxygenation without stopping treatment.

When predicting poor neurological outcomes in patients who survive CA, a multimodal approach has been recommended rather than a single test as an important component in predicting poor neurological outcomes with high predictive performance and high sensitivity at 100% specificity. The OHCA and CAHP scores are simple to use and useful, with 5–7 variables, and the predictive performance of poor neurological outcome has been reported to be 0.82 (95% CI 0.70–0.95) and 0.93 (95% CI 0.91–0.95), respectively, in original publications concerning these scores [4,5]. However, our study results showed that the AUCs of the OHCA and CAHP scores were 0.86 (95% CI 0.78–0.92) and 0.80 (95% CI 0.71–0.87), respectively, which were either higher or lower than those reported in the original publications. There are several possible explanations for this finding. Although our study included a limited number of patients compared to that of previous studies, it was more homogeneous, including only those who received TTM. In addition, the previous study determined the primary outcome determination time at hospital discharge and intensive care unit discharge, whereas in this study, the primary outcome was determined 3 months after ROSC.

In this study, we replaced the anoxia-related factor, i.e., no-flow time, with a similar objective variable to observe the change in prognosis prediction ability. The GWR of CT is a relatively easy and safe method most commonly used to predict prognosis before TTM [12,13]. However, Hong et al. reported that, in a prospective multicenter study of 512 patients, GWR that had been evaluated using brain CT only and conducted within 2 h after ROSC was not an independent factor for predicting poor neurological outcome [19]. In our study, the median time from ROSC to CT scan was 76.0 min (IQR, 41.0–117.0 min), and the prognostic predictive performance and sensitivity were the lowest among other objective methods evaluated (AUC 0.75; sensitivity, 13.3; specificity, 100%). Brain MRI is known to elucidate the brain’s structure in detail and to better predict hypoxia–ischemic brain injury than brain CT scanning [13]. Prognostic prediction methods using MRI before TTM have been reported using qualitative and quantitative analyses [15]. In this study, we used HSI on DWI as a qualitative method and PV 400 in ADC as a quantitative method. PV 400 was used in our study because Wijman et al. suggested that the percentage of brain volume with ADC values <400–450 × 10−6 mm^2^/s could differentiate between survival with independent and impaired functions [20], and our recent study found that PV 400 had the highest predictive power among the methods used [14]. In this study, all prognosis predictive performances using MRI were better than those using CT. Furthermore, HSI on DWI showed the highest predictive power and sensitivity (AUC 0.85; sensitivity, 69.2; specificity, 100%). In a recently published international guideline, the use of the biomarker NSE was recommended when predicting poor prognosis, in combination with other prognostic tests within 72 h after ROSC [21]. Vondrakova et al. reported that the AUC of the serum NSE level measured 6–30 h post-CA was 0.768 (sensitivity, 63.3%; specificity, 82.1%) for predicting poor neurological outcome 1 month after ROSC [22]. Son et al. reported that the AUC of the serum NSE level measured within 6 h after ROSC was 0.79 (sensitivity, 46.9%; specificity, 100%) [20,23]. Our study findings were similar to those of previous studies, as the AUC of NSE levels, taken early after ROSC, was 0.81 (sensitivity, 47.5%; specificity, 100%) [15].

Recently, Bae et al. reported the PROLOGUE model, which was shown to have excellent discrimination ability, compared with existing CA-specific risk scoring models, and had the highest predictive performance (AUC 0.93–0.94) among these scoring models for early prognosis prediction [8]. In that study and in another study, the major drawback of the OHCA and CAHP scores concerned the anoxia-related factor, namely, no-flow time [7,8]. This factor, in units of time, is challenging to calculate without the presence of a bystander witness (estimates are classified as categorical if no bystander witness is present), and one single minute could aggravate the prognosis by 7–10%, even though a logarithmic scale is applied [7,23]. However, the PROLOGUE model has low sensitivity (52.5%) at a specificity of 99.1% in predicting poor neurological outcomes and comprises more variables than the OHCA and CAHP scores, which limits its clinical application. Although our newly developed model used predictive values, our results indicated that the M-OHCA_HSI_ and M-CAHP_HSI_ models were not inferior to the PROLOGUE model in predicting the prognosis (AUC 0.96 and 0.97, respectively). In particular, our study showed higher sensitivity (74.5% and 82.4%, respectively) at 100% specificity. A possible explanation for the excellent performance in our study may be because all study cohorts that had HSI in DWI before TTM showed 100% poor neurological outcomes. Therefore, it is possible that this influenced the ability to predict prognosis and sensitivity. In addition, it is very rare for a patient to undergo an MRI immediately after ROSC. The priority after ROSC is to stabilize the patient and initiate TTM as soon as possible. For MRI examinations, TTM should not be postponed. However, our hospital has a suitable environment to perform MRI as quickly and safely as possible by minimizing movement to the MRI room and preparing an adequate monitoring system in the MRI room. As a result, the induction time to reach 33 degrees in our study was 357 min, which was no different than that seen in a large-scale randomized controlled trial [24,25].

This study had several limitations. First, this was a single-center retrospective study with a small number of patients; therefore, to increase the possibility of generalization of the results, it is necessary to develop a scoring system based on a larger number of patients. Furthermore, external validation should be performed in various contexts. Second, patients with CT, MRI, and NSE level measurements taken before TTM were included, and combinations of modified OHCA and CAHP models were evaluated. However, early (and high cost) MRI examinations are challenging to perform for CA survivors; hence, they are generally not easy to undertake. Third, bias due to a self-fulfillment prediction is possible because treating physicians were exposed to the results concerning CA etiology, laboratory findings, brain CT scans, DWI, and NSE levels in the included patients. However, in South Korea, WLST was not permitted before February 2018 unless a patient had been pronounced brain-dead and has been performed very rarely since then. No patients received WLST during TTM in this study. Finally, in brain DWI, reduced diffusion is known to be strongly associated with poor neurological outcomes, and these findings are most apparent between days 3 and 5 after ROSC [26]. Nevertheless, we emphasized that the brain MRIs performed initially following ROSC in CA patients provide valuable information, resulting in the improvement of the prognostic performance of both scoring systems.

## 5. Conclusions

In the OHCA and CAHP models, when no-flow time was replaced with HSI on DWI, PV 400 of ADC, GWR of CT, and serum NSE levels, the prediction and sensitivity of both models were further enhanced. HSI on DWI improved predictive power and sensitivity the most. To increase the possibility of generalization for our model, it is necessary to develop a scoring system based on a larger number of patients and additionally perform external validation in various contexts.

## Figures and Tables

**Table 1:** Baseline demographic data and arrest characteristics.

Characteristics	Cohort (*n* = 106)	Good Neurological Outcome (*n* = 45)	Poor Neurological Outcome (*n* = 61)	*p*-Value
Age, years, median (IQR)	57.0 (41.0–69.0)	57.0 (42.0–68.0)	57.0 (40.5–76.8)	0.773
Male gender, *n* (%)	78 (73.6)	37 (82.2)	41 (67.2)	0.193
Comorbidities, *n* (%)				0.544
Coronary artery disease	23 (21.7)	11 (24.4)	12 (19.7)	
Arrhythmia	15 (14.2)	5 (11.1)	10 (16.4)	
Atrial fibrillation	12 (11.3)	3 (6.7)	9 (14.8)	
WPW syndrome	1 (0.9)	1 (2.2)	0 (0)	
VPC	1 (0.9)	0 (0)	1 (1.6)	
1st degree AV block	1 (0.9)	1 (2.2)	0 (0)	
Cardiomyopathy	2 (1.9)	1 (2.2)	1 (1.6)	
Hypertrophic cardiomyopathy	2 (19)	1 (2.2)	1 (1.6)	
Heart failure	7 (6.6)	3 (6.7)	4 (6.6)	
Etiology of cardiac arrest, *n* (%)				0.863
Acute coronary syndrome	25 (23.6)	12 (26.7)	13 (21.3)	
Arrythmia	16 (15.1)	6 (13.3)	10 (16.4)	
Hypoxia	48 (45.3)	21 (46.7)	27 (44.3)	
Hyperkalemia	4 (3.8)	2 (4.4)	2 (3.3)	
Metabolic acidosis	2 (1.9)	2 (4.4)	2 (3.3)	
Anaphylaxis	1 (0.9)	1 (2.2)	0 (0)	
Pulmonary thromboembolism	1 (0.9)	0 (0)	1 (1.6)	
Unknown	9 (8.5)	3 (6.7)	6 (9.8)	
Arrest characteristics				
Witness, *n* (%),	69 (63.9)	36 (80)	33 (53.2)	0.004
Location of arrest, public place, *n* (%)	29 (26.9)	13 (28.9)	15 (24.2)	0.586
Bystander CPR, *n* (%)	77 (71.3)	39 (86.7)	38 (61.3)	0.004
Shockable rhythm, *n* (%)	30 (27.8)	24 (53.3)	5 (8.1)	<0.001
No flow time, min, median (IQR)	2.0 (0–13.0)	0.0 (0.0–5.0)	5.0 (0.0–22.0)	0.02
Low flow time, min, median (IQR)	20.0 (6.4–33.0)	15.0 (8.0–20.0)	29.0 (19.0–43.8)	<0.001
Epinephrine dose administered during CPR, mg, median (IQR)	2 (0–4)	0 (0–2)	3 (1.5–5)	<0.001
Laboratory parameters				
pH, median (IQR)	7.16 (7.00–7.32)	7.27 (7.08–7.35)	7.10 (6.97–7.30)	0.024
Lactate, mmol L^−1^, median (IQR)	7.75 (4.73–11.33)	7.70 (4.00–11.00)	7.80 (4.90–12.00)	0.050
Albumin, g dL^−1^, median (IQR)	3.3 (2.9–3.6)	3.4 (3.2–3.6)	3.2 (2.9–3.6)	0.015
Creatinine, mg dL^−1^, median (IQR)	1.26 (0.95–2.55)	1.26 (0.95–1.84)	1.27 (0.94–2.91)	0.350
NGAL, ng mL^−1^, median (IQR)	231.1 (100.8–677.7)	155.4 (78.3–451.6)	265.0 (130.7–683.0)	0.002
NSE, ng mL^−1^, median (IQR)				
Day 0	30.8 (23.3–58.0)	24.0 (18.0–30.1)	50.4 (29.4–73.3)	<0.001
Day 1	39.8 (24.3–116.0), 95 *	26.9 (20.6–35.8),40 *	82.6 (33.7–277), 55 *	<0.001
Day 2	35.1 (21.4–121.3), 88 *	22.4 (16.4–24.5), 39 *	97.2 (42.3–296.5), 49 *	<0.001
Day 3	36.6 (17.6–144.0), 83 *	18.3 (14.0–28.3), 38 *	113.4 (37.2–276.0), 45 *	<0.001
CK-MB, ng mL^−1^, median (IQR)	5.6 (2.6–9.8)	4.8 (1.9–8.4)	6.6 (3.4–12.0)	0.037
Troponin I, ng mL^−1^, median (IQR)	0.55 (0.06–52.8)	0.17 (0.03–34.6)	1.77 (0.10–273.00)	0.131
White blood cell, 10^3^ u L^−1^, median (IQR)	12.8 (8.8–17.6)	12.8 (8.7–17.4)	12.8 (8.8–18.7)	0.625
C-reactive protein, mg L^−1^, median (IQR)	0.6 (0.5–0.7)	0.5 (0.5–0.6)	0.6 (0.5–0.9)	0.249
Procalcitonin, ng mL^−1^, median (IQR)	0.22 (0.05–0.56)	0.05 (0.05–0.22)	0.30 (0.06–2.05)	0.003
Interleukin-6, pg mL^−1^, median (IQR)	411.8 (017.4–2012.5)	205.5 (57.8–513.6)	595.0 (129.8–5000.0)	0.013
TWA–PaCO_2_, mmHg, median (IQR)	38.8 (33.5–45.7)	41.4 (34.2–47.3)	37.9 (33.5–45.7)	0.334
ROSC to induction time at 33 °C, min (IQR)	357 (0.0-1140.0)	350.0 (120.0-767.0)	358.0 (0.0-1140.0)	0.680
Received intervention prior to TTM, *n* (%)				
Coronary angiography	31 (29.2)	12 (26.7)	19 (31.1)	0.670
Percutaneous coronary intervention	13 (12.3)	7 (15.6)	6 (9.8)	0.551
Brain image				
ROSC to CT time, min (IQR)	76.0 (41.0–117.0), 105 *	67.0 (35.0–93.0), 45 *	84.5 (49.8–134.3), 60 *	0.129
ROSC to MRI time, min (IQR)	156.0 (111.5–227.5), 89 *	131.0 (100.0–200.0), 37 *	165.0 (120.3–240.3), 52 *	0.294
GWR of CT, median (IQR)	1.21(1.11–1.29), 105 *	1.25 (1.20–1.31), 45 *	1.14 (1.06–1.24), 60 *	<0.001
HSI on DWI, number (%)	36 (33.3), 89 *	0 (0), 37 *	36 (69.2), 52 *	<0.001
PV 400 ** of ADC, median (IQR)	2.29 (0.32–4.18), 89*	0.38 (1.18–2.89), 37 *	3.41 (1.20–16.46), 52 *	<0.001
CA-specific risk score				
OHCA score	35.1 (23.4–56.0)	23.5 (16.9–29.3)	52.7 (38.0–61.2)	<0.001
CAHP score	181.0 (130.5–231.5)	130.5 (103.4–156.6)	217.5 (191.0–266.5)	<0.001
C-GRApH score	2.0 (2.0–3.0)	2.0 (1.0–3.0)	3.0 (2.0–3.0)	<0.001

IQR, interquartile range; WPW, Wolff–Parkinson–White; VPC, ventricular premature complexes; CPR, cardiopulmonary resuscitation; NGAL, neutrophil gelatinase associated lipocalin; NSE, neuron-specific enolase; TWA, time-weighted average; ROSC, return of spontaneous circulation; CT, computed tomography; MRI, magnetic resonance imaging; GWR, grey/white matter ratio; HSI, high signal intensity; DWI, diffusion-weighted image; PV, percentage of voxel; ADC, apparent diffusion coefficient; CA, cardiac arrest; OHCA, out-of-hospital cardiac arrest; CAHP, cardiac arrest hospital prognosis. * Number of patients included in the analysis; ** percentage of voxels below 400 × 10^−6^ mm^2^/s.

**Table 2:** A comparison of AUROC values for prediction scores, brain image, and serum NSE to predict poor neurological outcome at 3 months after ROSC.

	AUROC (95% CI)	Sensitivity (95% CI)	Specificity (95% CI)	PPV	NPV (95% CI)	*p*-Value for AUROC Comparison
Predicting CA-specific risk score						
OHCA	0.86 (0.78–0.92)	25.0 (14.7–37.9)	100.0 (92.0–100.0)	100.0	49.4 (45.8–53.1)	Reference
CAHP	0.80 (0.71–0.87)	5.0 (1.0–13.9)	100.0 (92.0-100.0)	100.0	43.6 (42.1–45.0)	0.17
C-GRApH	0.70 (0.60–0.78)	0.0 (0.0–6.0)	100.0 (92.0–100.0)		42.3 (42.3–42.3)	0.001
Brain image and serum NSE						
HSI on DWI, 89 *	0.85 (0.75–0.91)	69.2 (54.9–81.3)	100.0 (90.5–100.0)	100.0	69.8 (60.6–77.7)	Reference
PV 400 ** of ADC, 89 *	0.78 (0.68–0.86)	40.4 (27.0–54.9)	100.0 (90.5–100.0)	100.0	54.4 (48.8–59.9)	0.19
GWR of CT, 105 *	0.75 (0.66–0.83)	13.3 (5.9–24.6)	100.0 (92.1–100.0)	100.0	46.4 (43.9–48.9)	0.09
NSE, 106 *	0.81 (0.73–0.88)	47.5 (34.6–60.7)	100.0 (92.1–100.0)	100.0	58.4 (52.5–64.1)	0.55

AUROC, the area under the receiver operating characteristic curve; NSE, neuron-specific enolase; ROSC, return of spontaneous circulation; PPV, positive predictive value; NPV, negative predictive value; CA, cardiac arrest; OHCA, out-of-hospital cardiac arrest; CAHP, cardiac arrest hospital prognosis. HSI, high signal intensity; DWI, diffusion-weighted image; PV, percentage of voxel; ADC, apparent diffusion coefficient; GWR, grey/white matter ratio; CT, computed tomography. * Number of patients included in the analysis; ** percentage of voxels below 400 × 10^−6^ mm^2^/s.

**Table 3:** A comparison of prognostic performance using the modified OHCA (out-of-hospital cardiac arrest) and CAHP (cardiac arrest hospital prognosis) models.

Provability Values	AUROC (95% CI)	Sensitivity (95% CI)	Specificity (95% CI)	PPV	NPV (95% CI)	*p*-Value for AUROC Comparison
Modified OHCA model, 106 *	0.89 (0.81–0.94)	33.3 (21.7–46.7)	100.0 (92.0–100.0)	100.0	52.4 (47.9–56.8)	Reference
Modified OHCA (HSI on DWI), 89 *	0.96 (0.90–0.99)	74.5 (60.4–85.7)	100.0 (90.5–100.0)	100.0	74.0 (64.0–82.0)	0.01
Modified OHCA (PV 400 of ADC), 89 *	0.93 (0.85–0.97)	49.0 (34.8–63.4)	100.0 (90.5–100.0)	100.0	58.7 (52.1–65.1)	0.27
Modified OHCA (GWR of CT), 105 *	0.92 (0.85–0.97)	35.6 (23.6–49.1)	100.0 (92.0–100.0)	100.0	53.7 (48.9–58.3)	0.09
Modified OHCA(NSE), 106 *	0.92 (0.85–0.96)	54.4 (40.7–67.6)	100.0 (91.6–100.0)	100.0	61.8 (54.9–68.2)	0.05
Modified CAHP model, 106 *	0.90 (0.82–0.95)	30.0 (18.8–43.2)	100.0 (92.0–100.0)	100.0	51.2 (47.0–55.3)	Reference
Modified CAHP (HSI on DWI), 89 *	0.97 (0.91–0.99)	82.4 (69.1–91.6)	100.0 (90.5–100.0)	100.0	80.4 (69.4–88.1)	0.01
Modified CAHP (PV 400 ** of ADC), 89 *	0.93 (0.86–0.97)	60.8 (46.1–74.2)	100.0 (90.5–100.0)	100.0	64.9 (56.8–72.2)	0.13
Modified CAHP (GWR of CT), 105 *	0.92 (0.85–0.97)	18.6 (9.7–30.9)	100.0 (92.0–100.0)	100.0	47.8 (44.8–50.9)	0.18
Modified CAHP (NSE), 106 *	0.91 (0.83–0.96)	57.9 (44.1–70.9)	100.0 (91.6–100.0)	100.0	63.6 (56.3–70.4)	0.64

AUROC, the area under the receiver operating characteristic curve; PPV, positive predictive value; NPV, negative predictive value; HSI, high signal intensity; DWI, diffusion-weighted image; PV, percentage of voxel; ADC, apparent diffusion coefficient; GWR, grey/white matter ratio; CT, computed tomography; NSE, neuron-specific enolase. * Number of patients included in the analysis; ** percentage of voxels below 400 × 10^−6^ mm^2^/s.

## Data Availability

The data presented in this study are available on request from the corresponding author. The data are not publicly available due to privacy or ethical restriction.

## Abbreviations

ADC	apparent diffusion coefficient
AUROC	area under the receiver operating characteristic curve
CA	cardiac arrest
CAHP	cardiac arrest hospital prognosis
CI	confidence interval
CK-MB	creatinine kinase myocardial band
CN	caudate nucleus
CPC	cerebral performance category
CPR	cardiopulmonary resuscitation
CNUH	Chungnam National University Hospital
CT	computed tomography
DWI	diffusion-weighted imaging
ECMO	extracorporeal membrane oxygenation
FMRIB	functional magnetic resonance imaging of the brain
GWR	grey/white matter ratio
HIS	high-signal intensity
IQR	interquartile range
MRI	magnetic resonance imaging
NGAL	neutrophil gelatinase-associated lipocalin
NSE OHCA	neuron-specific enolase out-of-hospital cardiac arrest
P	putamen
PIC	posterior limb of the internal capsule
PV	percentage voxels
ROC	receiver operating curve
ROSC	return of spontaneous circulation
T	thalamus
TTM	targeted temperature management
TWA	time-weighted average
WLST	withdrawal of life-sustaining treatment
